# Screening older adults for amnestic mild cognitive impairment and early-stage Alzheimer’s disease using upper-extremity dual-tasking

**DOI:** 10.1038/s41598-019-46925-y

**Published:** 2019-07-29

**Authors:** Nima Toosizadeh, Hossein Ehsani, Christopher Wendel, Edward Zamrini, Kathy O’ Connor, Jane Mohler

**Affiliations:** 1 Department of Biomedical Engineering, University of Arizona, Tucson, AZ USA; 2 Arizona Center on Aging (ACOA), Department of Medicine, University of Arizona, College of Medicine, Tucson, AZ USA; 3 Division of Geriatrics, General Internal Medicine and Palliative Medicine, Department of Medicine, University of Arizona, Tucson, AZ USA; 4 Banner Sun Health Research Institute, Sun City, AZ USA; 5 Banner Alzheimer’s Institute, University of Arizona, Tucson, AZ USA; 6 Department of Neurology, University of Utah, Salt Lake City, UT USA

**Keywords:** Alzheimer's disease, Sensors and probes

## Abstract

The purpose of the current study was to develop an objective tool based on dual-task performance for screening early-stage Alzheimer’s disease (AD) and mild cognitive impairment (MCI of the Alzheimer’s type). Dual-task involved a simultaneous execution of a sensor-based upper-extremity function (UEF) motor task (normal or rapid speed) and a cognitive task of counting numbers backward (by ones or threes). Motor function speed and variability were recorded and compared between cognitive groups using ANOVAs, adjusted for age, gender, and body mass index. Cognitive indexes were developed using multivariable ordinal logistic models to predict the cognitive status using UEF parameters. Ninety-one participants were recruited; 35 cognitive normal (CN, age = 83.8 ± 6.9), 34 MCI (age = 83.9 ± 6.6), and 22 AD (age = 84.1 ± 6.1). Flexion number and sensor-based motion variability parameters, within the normal pace elbow flexion, showed significant between-group differences (maximum effect size of 1.10 for CN versus MCI and 1.39 for CN versus AD, *p* < 0.0001). Using these parameters, the cognitive status (both MCI and AD) was predicted with a receiver operating characteristic area under curve of 0.83 (sensitivity = 0.82 and specificity = 0.72). Findings suggest that measures of motor function speed and accuracy within a more practical upper-extremity test (instead of walking) may provide enough complexity for cognitive impairment assessment.

## Introduction

Cognitive impairment is a critical health problem with an increasing prevalence because of the population aging^1^. It is estimated that by 2040, the number of elders living with dementia will surpass nine million in the US, roughly 170% increase compared to 2001^2^. Among different types of dementia, Alzheimer’s disease (AD) is the most common type, which influences the lives of up to 7% of the elderly population in the US and globally^3^. Early dementia screening provides an opportunity to begin secondary prevention, as well as planning for future care, safety concerns, and financial and legal arrangements, while decision-making capacity remains^4^. Unfortunately, many providers are reluctant to screen for dementia resulting in less than half of patients with AD having ever received a formal diagnosis^5^.

The current research was founded based on the fact that simultaneous declines in motor and cognitive performance occur with aging^6–8^. In many age-related neurodegenerative diseases, and more specifically in Alzheimer’s disease, compensatory processes in cortical and subcortical brain regions allow maintenance of motor and cognitive performance^9^. Assessing deficits in dual-tasking, therefore, can provide a powerful tool for screening cognitive impairments. Gait has been commonly used as the motor task component in dual-task assessments. Poor dual-task gait performance has been significantly correlated with decreased executive and neuropsychological function, and demonstrated to be associated with AD or even mild cognitive impairment (MCI)^10–12^. However, many older adults have mobility impairments, and many clinics lack adequate space to safely perform gait measures.

We have previously developed and validated an upper-extremity function (UEF) test to assess slowness, weakness, exhaustion, and flexibility, with the main purpose of frailty assessment among older adults^13–15^. The UEF test integrates low-cost sensors and data acquisition system (as low as $200), the physical assessment (including preparation/calibration) is easily performed in less than five minutes, and the post-processing is performed in less than two minutes. In previous work, we have determined strong correlations between UEF and gait speed^14^ and six-minute walk distance^16^. Further, we have compared the UEF dual-task performance (simultaneous performance of UEF and a cognitive task of counting backward) with gait dual-task performance, as well as the Montreal Cognitive Assessment (MoCA) test^17,18^. Within this work, a significant association has been observed between UEF speed parameters and gait speed within the dual-task trials. Also, significant correlations have been shown between MoCA and UEF dual-task motor function speed and variability.

In continuation of previous work, the purpose of the current study was to investigate the capability of UEF for discriminating clinically diagnosed elders with cognitive impairment and to develop an index for screening AD and MCI. The hypotheses were: (1) UEF dual-task performance would be significantly different between cognitive groups; (2) UEF dual-task performance would decrease with cognitive task difficulty, and this response would impact cognitive impairment predictions; and (3) instructing participants to perform the motor task component consistently (instead of rapidly) would improve the cognitive impairment predictions. If found to be valid, the proposed UEF tool would provide several advantages compared to currently available cognitive impairment screening tools such as MoCA, including^19–23^: (1) providing an objective score based on motor function; (2) capability for routinely assessment with minimum learning effects; (3) being less influenced by education and language, since the actual scoring is based on motor function performance rather than a cognitive task; and (4) providing a simple assessment protocol, without requiring the global judgment of the examiner (e.g., as it is required for clock-drawing test).

## Methods

### Participants

Participants were recruited from the Banner Sun Health Research Institute (BSHRI) from September 2017 to May 2018. Eligible participants from the BSHRI cohort were vetted by the clinic director (KO) to assure eligibility for participation. Then they received a pre-clinic phone call or an in-clinic opportunity to discuss the study and enrollment process. Written informed consent according to the principles expressed in the Declaration of Helsinki^24^ was then obtained from eligible subjects before participation. The study was approved by the University of Arizona Institutional Review Board. Participants were selected and stratified into three categories of cognitive status including cognitive normal (CN), MCI of the Alzheimer’s type, and early-stage AD. The clinical diagnosis of cognitive status happened within a time-window of six months prior to study measurements, based on the National Institute of Aging – Alzheimer’s Association (NIA-AA) criteria^25,26^. Cognitive groups were frequency matched (controlled distributions) on age categories and gender to assure equivalent age and gender distribution. Inclusion criteria were: (1) age of 65 years or older; (2) ability to understand study instructions; and (3) English language proficiency. Exclusion criteria were: (1) diagnosed diseases associated with severe motor performance deficits including stroke or Parkinson’s disease; (2) severe speech disorders; and (3) severe upper-extremity disorders, including elbow bilateral fractures or rheumatoid arthritis.

### Clinical measures

Neuropsychological tests were conducted as a part of prior diagnostic assessments (within a time-window of six months prior to study measurements) or collected during the study data collection. Of note, the association between UEF and neuropsychological tests is beyond the scope of the current paper and is investigated in another study. Here, only associations between UEF dual-task performance with MoCA^27^ and the Mini–Mental State Examination (MMSE)^28^ were investigated.

In addition to neuropsychological tests, we collected the following clinical measures: (1) frailty (the Fried Index)^29^; (2) comorbidity (Charlson comorbidity score (CCI))^30^; and (3) depression Patient Health Questionnaire (PHQ-9)^31^. These three clinical measures were added as potential confounding factors in dual-task performance. Physical frailty was assessed to check differences in function between groups, since the proposed cognitive measurement is based on motor function performance. Comorbidity was measured as it is associated with an increased risk of cognitive impairment^32^. Finally, we assessed the level of depression at the time of the measurement, to take into account its potential confounding effect on dual-task performance. We measured depression, since results from a recent systematic review revealed significant cognitive deficits in executive function, memory, and attention in patients with depression, compared to healthy individuals^33^.

### UEF cognitive assessment protocol and outcomes

**Figure 1:**
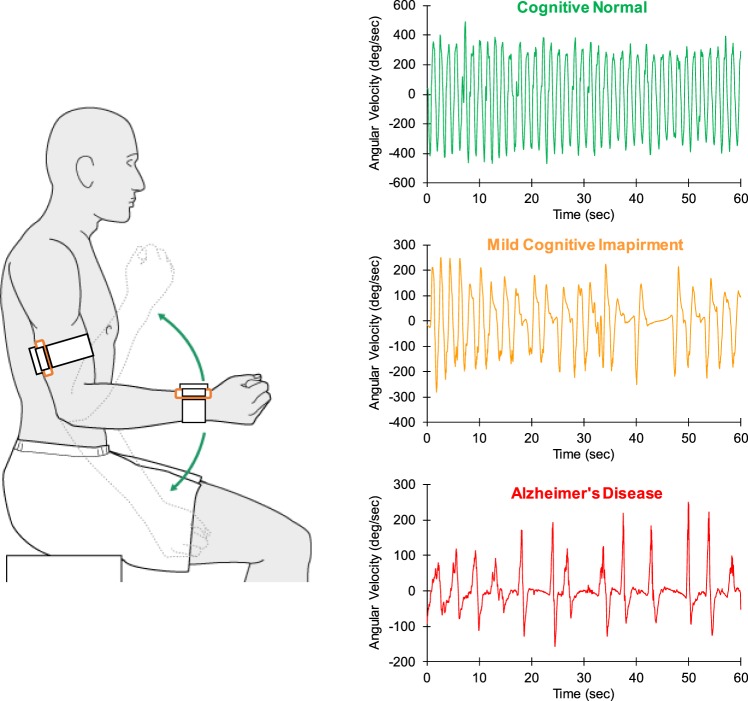
UEF procedure for identifying cognitive impairment: Two gyroscopes are attached to the wrist and upper-arm of the dominant arm to measure the elbow angular velocity. Data are presented for 60-second dual-task motor performance while counting numbers backward by threes, for participants from three cognitive groups (the same age range 72–74).

After completing questionnaires, each participant performed the UEF elbow flexion test with the dominant arm. The UEF task consists of six trials, including a full-combination of two speed of elbow flexion (rapid and normal self-selected pace) and three cognitive conditions (no counting, counting backward by ones, and counting backward by threes starting from a random two-digit number) (Fig. 1). Within the rapid pace, the participant flexed and extended the elbow as quickly as possible in 20 seconds. Within the self-selected pace, participants performed the task as consistently as possible in 60 seconds. We considered speed and consistency to represent delay and accuracy aspects of dual-tasking, as they have been related to cognitive aging^7^. The order of six UEF trials was randomized to minimize confounding fatigue and learning effects. To better represent the natural environment for daily activities, there was no instruction to prioritize either the physical or the counting task^12,34,35^. The cognitive task of counting numbers was selected as it involves working memory^36^ and, therefore, is related to executive functioning, compared to other tasks such as naming objects/animals^12,37,38^. Counting is also a rhythmic task that greatly interferes with the rhythmicity of repetitive elbow flexion^38,39^.

Before measurements, participants were equipped with the UEF system. A tri-axial wearable gyroscope and accelerometer sensor (sample frequency = 100 Hz, BioSensics LLC, Boston, MA, USA), was attached to the upper arm near the biceps and one to the wrist, both on one arm, using a band attached with Velcro, to estimate three-dimensional angular velocity of the upper-arm and forearm, and ultimately elbow flexion (Fig. 1).

Several outcome measures representing kinematics of the elbow flexion were derived using angular velocity. To extract the outcome measure, the angular velocity signal from the sensors were filtered to remove drifting (first order high pass butter-worth filter with a cutoff of 2.5 Hz), and using a peak detection algorithm maximum and minimums of the angular velocity signal, and subsequently, elbow flexion cycles were detected. Accordingly, UEF outcome measures were calculated, including: (1) speed; (2) rise time; (3) range of motion (ROM); (4) speed variability; (5) ROM variability; (6) flexion variability; and (7) flexion number (for definition of each parameter see Table 1). Of note, all the above outcomes can be calculated using an in-home MATLAB app, within less than 1 minute. These parameters were selected to present alterations in motor function execution speed and accuracy within dual-tasking, based on our previous investigations and gait dual-task studies among dementia patients^12,17,18,40,41^. Using these parameters, changes in motor function due to the cognitive task were determined, which included: (1) agility (speed, rise time, and flexion number, which are analogous to gait speed, stride time, and cadence); (2) flexibility (ROM, which is analogous to step length); and (3) variability (speed, ROM, and flexion variability, which are analogous to gait speed, step length, and gait cycle time variability). To assess changes in the above parameters in an individual’s performance from a single to a dual-task, dual-task “cost” was measured for each parameter as the percentage of change within two conditions.

**Table 1:** UEF parameter definitions.

UEF Parameters		Definition
Agility	Speed	Mean value of the elbow angular velocity range (maximum minus minimum speed)
	Rise time	Mean value of the time required to reach the maximum angular velocity
	Flexion number	Total number of flexion/extensions
Flexibility	ROM	Mean value of the elbow flexion range
Variability	Speed variability	Coefficient of Variation (COV) of the angular velocity range
	ROM variability	COV of the flexion angle range
	Flexion variability	COV of time distances between consecutive angular velocity peaks

UEF: upper-extremity function; ROM: range of motion.

To assess the secondary cognitive task performance (i.e., counting numbers), the number of correctly counted numbers and the number of mistakes within each arm test were considered as outcomes. These parameters represent the speed and accuracy of the secondary task within the dual-task condition^42^.

### Statistical analysis and cognitive index development

Analysis of variance (ANOVA) models were used to evaluate the differences in all of the demographic and clinical parameters between three cognitive groups (i.e., CN, MCI, and AD), except gender, comorbidity, and frailty prevalence; chi-square *χ*^2^ tests were used to assess gender and comorbidity categories differences, and the Fisher’s exact test was used to assess frailty prevalence differences between cognitive groups.

UEF parameters for all six conditions as well as UEF dual-task cost variables were compared between groups using ANOVA models (CN vs. MCI, MCI vs. AD, and CN vs AD); age, gender, and body mass index (BMI) were considered as covariates and Cohen’s effect size (*d*) was estimated. Post-hoc Tukey’s honest significant difference tests were performed for three pairwise comparisons between the cognitive groups. Of note, age, gender, and BMI were selected as adjusting variables, since they have been previously associated with AD and, in general, dementia. Women are expected to have a higher risk of developing AD compared to men^43^. BMI as a common measure of nutritional status was considered as an adjusting variable, since BMI < 25 kg/m2 is associated with the risk of moderate-severe cognitive impairment^44^. Further, there is evidence that excess weight may adversely affect executive function, attention, memory, and the overall cognition^45^.

Two types of UEF cognitive indexes were developed similar to previous work^15^. The first index (UEF cognitive categorical index) was developed to predict three cognitive groups including CN, MCI, and AD (per participant categories within this study). Multivariable ordinal logistic models with the cognitive status as the dependent variable, and UEF parameters plus demographic information (i.e., age, gender, and BMI) as independent variables were used to develop the UEF categorical index (see Supplementary Information 1 for details regarding UEF categorical cognitive index and the cross-validation process). We tested the proportional odds assumption for ordinal logistic regression models using an approximate likelihood-ratio test of whether the coefficients are equal across categories.

To develop the second UEF index (UEF cognitive score) parameters selected from the categorical index were used, following methods developed for the Framingham cardiovascular risk score^46^. Cut-offs and weight for each of UEF parameter were determined based on mean values for each frailty group and parameter estimates from the categorical logistic models. The UEF cognitive score (from 0: CN to 1: AD) for a given participant was defined as the sum of points corresponding to performance results from UEF dual-task test (see Supplementary Information 2 for details regarding UEF cognitive score).

Finally, the association between UEF score with MoCA and MMSE were assessed using the Pearson correlation (and Spearman’s rank if not normally distributed).

The datasets generated during and/or analyzed during the current study are available from the corresponding author on reasonable request.

### Power calculation

The power calculation for this study was based on our previous work among 10 cognitively impaired and 57 cognitively intact participants^18^. We observed comparisons of impaired vs intact for UEF dual-task speed 864.81 ± 299.91 vs 455.05 ± 237.20 deg/s, UEF dual-task speed variability 16.30 ± 8.24 vs 41.75 ± 18.52%, and UEF dual-task ROM 100.58 ± 29.26 vs 73.60 ± 28.91 deg. Based on the variation observed in dual-task performance measures, a sample size of 30 per group (alpha = 0.05) was expected to provide 80% power to detect a difference of: (1) 200 deg/s for speed; (2) 11% for speed variability; and (3) 21 deg for ROM. Because these detectable differences were substantially below those observed in the pilot data, we expected to have adequate power to detect differences for at least these parameters between MCI and controls and between early-stage AD and controls. Of note, we observed significant differences between these parameters in our study; however, different UEF parameters emerged as the most explanatory independent variables and eventually were used in the UEF indexes.

## Results

### Participants

Ninety-one participants were recruited, including 35 CN (age = 83.8 ± 6.9), 34 MCI (age = 83.9 ± 6.6), and 22 AD (age = 84.1 ± 6.1) older adults. Of note, no participant was excluded because of incapability to perform the UEF test. Age, gender, height, and weight were not significantly different between these groups (*p* > 0.41, Table 2). Frailty status, and comorbidity and depression scores were also not significantly different between cognitive groups (*p* > 0.08, Table 2).

**Table 2:** Differences in demographic and clinical measures among cognitive groups. A significant difference between groups is highlighted in bold.

Demographic Information	CN (n = 35)	MCI (n = 34)	AD (n = 22)	*p*-value^†^ (Effect size^‡^)
Male, n (% of the group)	13 (37%)	16 (47%)	11 (50%)	0.57 (0.11)
Age, year (SD)	83.83 (6.92)	83.88 (6.57)	84.05 (6.12)	0.99 (0.01)
Height, cm (SD)	167.83 (9.97)	170.32 (9.47)	166.93 (11.06)	0.41 (0.14)
Weight, kg (SD)	71.15 (15.67)	71.09 (17.45)	67.02 (12.46)	0.57 (0.11)
Body mass index, kg/m^2^ (SD)	25.27 (5.26)	24.37 (5.36)	23.96 (3.54)	0.59 (0.11)
Education, year (SD)	16.00 (2.47)	14.70 (2.89)	14.48 (2.89)	0.07 (0.25)
**Clinical Measures**	**CN** (**n** = **35**)	**MCI** (**n** = **34**)	**AD** (**n** = **22**)	***p*-value**^**†**^ (**Effect size**^**‡**^)
Montreal cognitive assessment, 0–30 (MoCA) (SD)	26.77 (2.46)	21.94 (2.63)	17.29 (1.59)	**<0.0001 (1.58)**
Mini-Cog, 0–5 (SD)	4.43 (0.85)	3.24 (1.33)	1.57 (1.08)	**<0.0001 (1.01)**
Mini-mental state examination, 0–30 (MMSE) (SD)	28.51 (1.56)	26.54 (2.38)	21.33 (4.20)	**<0.0001 (1.07)**
Frailty category, n (%)				0.24 (0.18)
Non-frail	12 (34.3%)	12 (35.3%)	3 (13.6%)	
Pre-frail	19 (54.4%)	16 (47.1%)	16 (72.7%)	
Frail	3 (8.6%)	6 (17.7%)	2 (9.1%)	
Charlson-Deyo Comorbidity Index >1, n (%)	21 (60.0%)	20 (58.8%)	7 (31.8%)	0.08 (0.24)
Patient Health Questionnaire (PHQ-9), 0–27 (SD)	1.74 (2.74)	2.61 (2.95)	2.14 (2.61)	0.45 (0.14)

CN: cognitive normal; MCI: mild cognitive impairment; AD: Alzheimer’s disease.

SD: standard deviation.

^†^One-way analysis of variance (ANOVA) *F*-test, except chi-square for gender and Charlson-Deyo, and Fisher’s exact for frailty.

^‡^Cohen’s *f* for ANOVA, and Cramér’s V for Chi-square and Fisher’s tests.

### UEF dual-task performance among cognitive groups

**Figure 2:**
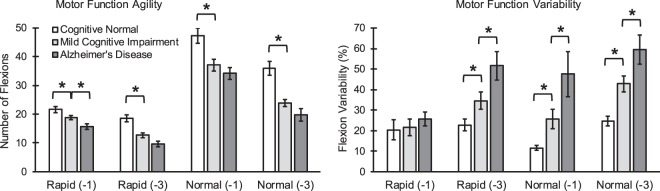
Differences in upper-extremity motor function agility and variability within dual-task conditions (rapid and normal self-selected flexion speed in combination with counting numbers backward by ones and threes). Significant differences between cognitive groups are highlighted with asterisk symbols.

ANOVA results showed significant differences in executing motor function between cognitive groups, especially within dual-task trials (Fig. 2). On average among two dual-task difficulties and two motor task conditions, flexion number demonstrated the largest overall effect size for between CN and MCI group comparisons (average effect size = 0.83 ± 0.23; max effect size = 1.10), and between CN and AD group comparisons (average effect size = 1.20 ± 0.18; max effect size = 1.38). For MCI and AD group comparisons, ROM variability showed, on average between all conditions, the largest overall effect size (average effect size = 0.55 ± 0.16; max effect size = 0.69).

Within the single-task trials with no cognitive task, significant differences were only observed in the agility parameters of elbow flexion, and not flexibility and variability (Tables 3 and 4). The overall effect sizes for all between group comparisons were 0.40 ± 0.20, 0.14 ± 0.08, and 0.25 ± 0.16 for agility, flexibility, and variability parameters, respectively, within the single-task condition. Differences between UEF parameters among the three cognitive groups were more noticeable within dual-task conditions; significant differences were observed for all motor function categories, including agility, flexibility, and variability of elbow flexion. The overall effect sizes for all between group comparisons were 0.57 ± 0.30, 0.23 ± 0.13, and 0.46 ± 0.30 for agility, flexibility, and variability parameters, respectively, within the dual-task counting backward by ones. The corresponding effect size values were further increased to 0.70 ± 0.42, 0.38 ± 0.15, and 0.67 ± 0.32 within the dual-task counting by threes.

**Table 3:** Differences in UEF performance among cognitive groups – Results from ANOVA for rapid flexion tests. All comparisons were adjusted with age, gender, and body mass index (BMI). A significant difference between groups is highlighted in bold.

Variable	UEF average values and (standard deviations)			*p*-value (Effect size^†^)		
No Counting	CN (n = 35)	MCI (n = 34)	AD (n = 22)	CN vs. MCI	MCI vs. AD	CN vs. AD
Speed, deg/sec	895.82 (270.43)	826.60 (296.81)	710.21 (230.28)	0.2013 (0.24)	0.1014 (0.44)	**0.0016 (0.74)**
Rise time, msec	228.65 (62.97)	238.91 (69.14)	289.10 (97.5)	0.4165 (0.16)	**0.0142 (0.59)**	**0.0024 (0.74)**
Flexion number, n	26.23 (8.97)	24.21 (6.99)	20.71 (6.75)	0.1871 (0.25)	0.0950 (0.48)	**0.0063 (0.70)**
ROM, deg	108.21 (29.75)	105.97 (37.99)	100.26 (28.88)	0.7358 (0.06)	0.4992 (0.18)	0.2476 (0.27)
Speed variability, %	9.03 (4.32)	11.58 (6.39)	11.56 (5.40)	0.0642 (0.47)	0.9852 (0.01)	0.0520 (0.52)
ROM variability, %	10.50 (5.87)	13.77 (8.88)	12.37 (10.06)	0.0946 (0.44)	0.5598 (0.15)	0.2514 (0.23)
Flexion variability, %	9.16 (9.05)	7.93 (4.15)	9.41 (7.16)	0.3974 (0.18)	0.4682 (0.26)	0.9422 (0.03)
**Backward by ones**	**CN (n** = **35)**	**MCI (n** = **34)**	**AD (n** = **22)**	**CN vs. MCI**	**MCI vs. AD**	**CN vs. AD**
Speed, deg/sec	771.32 (233.50)	711.13 (254.40)	573.08 (182.96)	0.1817 (0.25)	**0.0198 (0.62)**	**0.0002 (0.95)**
Rise time, msec	280.66(112.31)	296.80 (88.72)	356.59 (125.01)	0.4747 (0.16)	**0.0442 (0.55)**	**0.0232 (0.64)**
Flexion number, n	21.66 (6.41)	18.85 (4.26)	15.68 (4.52)	**0.0194 (0.55)**	**0.0264 (0.74)**	**0.0003 (1.08)**
ROM, deg	103.35 (33.31)	106.98 (36.42)	94.31 (30.20)	0.7763 (0.13)	0.1501 (0.43)	0.2221 (0.29)
Speed variability, %	12.24 (5.28)	12.01 (3.71)	14.30 (5.80)	0.8797 (0.05)	0.0923 (0.47)	0.2047 (0.38)
ROM variability, %	11.07 (5.49)	11.19 (5.62)	13.78 (9.16)	0.9282 (0.03)	0.1578 (0.35)	0.1652 (0.36)
Flexion variability, %	20.38 (28.73)	21.47 (23.67)	25.79 (15.88)	0.9287 (0.04)	0.5431 (0.24)	0.5709 (0.25)
Counted numbers, n	20.66 (7.06)	19.53 (7.50)	13.41 (7.37)	0.4922 (0.16)	0.1566 (0.35)	**0.0011 (1.01)**
Counting mistakes, n	0.34 (0.80)	0.53 (0.86)	1.00 (1.72)	0.4660 (0.23)	0.0908 (0.49)	**0.0047 (0.82)**
**Backward by threes**	**CN (n** = **35)**	**MCI (n** = **34)**	**AD (n** = **22)**	**CN vs. MCI**	**MCI vs. AD**	**CN vs. AD**
Speed, deg/sec	611.59 (207.17)	586.68 (282.71)	454.82 (165.68)	0.4612 (0.10)	**0.0259 (0.57)**	**0.0009 (0.84)**
Rise time, msec	346.48 (159.23)	542.23 (285.06)	695.86 (450.43)	**0.0035 (0.85)**	**0.0495 (0.41)**	**<0.0001 (1.03)**
Flexion number, n	18.54 (7.74)	12.65 (4.93)	9.59 (4.95)	**<0.0001 (0.91)**	0.0615 (0.61)	**<0.0001 (1.38)**
ROM, deg	94.03 (27.58)	105.99 (40.04)	82.21 (34.87)	0.2185 (0.35)	**0.0082 (0.63)**	**0.0003 (0.38)**
Speed variability, %	16.72 (7.80)	15.84 (5.32)	25.01 (16.64)	0.7809 (0.13)	**0.0011 (0.75)**	**0.0137 (0.64)**
ROM variability, %	12.49 (8.94)	13.53 (6.83)	30.27 (34.63)	0.7800 (0.13)	**0.0013 (0.67)**	**0.0060 (0.70)**
Flexion variability, %	22.71 (17.03)	34.59 (24.15)	51.57 (32.97)	**0.029 (0.57)**	**0.0104 (0.59)**	**<0.0001 (1.10)**
Counted numbers, n	9.17 (3.78)	7.29 (3.92)	5.50 (4.23)	**0.0302 (0.49)**	0.0533 (0.44)	**0.0001 (0.91)**
Counting mistakes, n	0.66 (1.11)	0.94 (1.63)	0.91 (1.41)	0.4238 (0.20)	0.8378 (0.02)	0.4611 (0.20)

UEF: upper-extremity function; ANOVA: analysis of variance; ROM: range of motion.

CN: cognitive normal; MCI: mild cognitive impairment; AD: Alzheimer’s disease.

^†^Cohen’s *d* for ANOVA.

**Table 4:** Differences in UEF performance among cognitive groups – Results from ANOVA for normal self-selected pace flexion tests. All comparisons were adjusted with age, gender, and body mass index (BMI). A significant difference between groups is highlighted in bold.

Variable	UEF average values and (standard deviations)			*p*-value (Effect size)		
No Counting	CN (n = 35)	MCI (n = 34)	AD (n = 22)	CN vs. MCI	MCI vs. AD	CN vs. AD
Speed, deg/sec	538.38 (151.41)	490.74 (153.36)	464.43 (126.28)	0.1744 (0.31)	0.5598 (0.19)	**0.0494 (0.53)**
Rise time, msec	375.11 (133.65)	451.09 (215.52)	421.66 (142.54)	0.0995 (0.43)	0.5736 (0.16)	0.2230 (0.33)
Flexion number, n	46.69 (14.71)	41.52 (20.69)	39.41 (13.28)	0.2434 (0.29)	0.6023 (0.12)	0.0594 (0.52)
ROM, deg	102.43 (31.69)	99.79 (32.00)	97.86 (26.43)	0.5615 (0.10)	0.8462 (0.07)	0.4005 (0.17)
Speed variability, %	9.28 (3.47)	9.88 (4.75)	10.81 (4.77)	0.7355 (0.14)	0.4188 (0.20)	0.1773 (0.37)
ROM variability, %	9.73 (12.49)	9.87 (6.82)	8.49 (4.61)	0.8789 (0.01)	0.5895 (0.24)	0.8869 (0.13)
Flexion variability, %	7.28 (2.94)	11.11 (13.83)	9.06 (4.50)	0.1061 (0.38)	0.4005 (0.20)	0.0937 (0.47)
**Backward by ones**	**CN (n** = **35)**	**MCI (n** = **34)**	**AD (n** = **22)**	**CN vs. MCI**	**MCI vs. AD**	**CN vs. AD**
Speed, deg/sec	526.61 (163.27)	461.04 (167.23)	454.54 (136.79)	0.0639 (0.40)	0.8339 (0.05)	0.0514 (0.48)
Rise time, msec	363.19 (120.16)	435.86 (134.93)	470.11 (125.51)	**0.0130 (0.57)**	0.3800 (0.27)	**0.0012 (0.87)**
Flexion number, n	47.31 (15.45)	37.09 (12.00)	34.23 (9.90)	**0.0015 (0.75)**	0.2959 (0.25)	**0.0006 (1.01)**
ROM, deg	99.82 (31.39)	97.38 (34.94)	91.65 (27.26)	0.5859 (0.07)	0.4515 (0.18)	0.1873 (0.28)
Speed variability, %	10.45 (3.40)	12.51 (4.90)	14.95 (4.79)	**0.0382 (0.49)**	**0.0172 (0.50)**	**0.0001 (1.08)**
ROM variability, %	8.40 (4.20)	10.64 (5.83)	14.50 (9.22)	0.1187 (0.44)	**0.0071 (0.50)**	**0.0004 (0.85)**
Flexion variability, %	11.50 (7.02)	25.58 (27.85)	47.57 (51.47)	**0.0432 (0.69)**	**0.0086 (0.53)**	**<0.0001 (0.99)**
Counted numbers, n	44.83 (14.42)	50.38 (11.00)	50.68 (13.73)	0.0740 (0.43)	0.7126 (0.02)	0.0808 (0.42)
Counting mistakes, n	0.66 (1.25)	1.03 (1.85)	2.09 (2.07)	0.4145 (0.23)	**0.0346 (0.54)**	**0.0028 (0.84)**
**Backward by threes**	**CN (n** = **35)**	**MCI (n** = **34)**	**AD (n** = **22)**	**CN vs. MCI**	**MCI vs. AD**	**CN vs. AD**
Speed, deg/sec	446.98 (176.85)	399.39 (143.88)	397.85 (157.01)	0.0858 (0.30)	0.8925 (0.01)	0.1923 (0.29)
Rise time, msec	483.28 (183.20)	767.56 (410.68)	886.30 (432.85)	0.0009 (0.89)	0.2081 (0.28)	**<0.0001 (1.21)**
Flexion number, n	35.97 (14.43)	23.88 (7.53)	19.73 (10.48)	<0.0001 (1.10)	0.1569 (0.48)	**<0.0001 (1.34)**
ROM, deg	93.74 (31.77)	99.42 (32.86)	84.92 (29.51)	0.5943 (0.19)	0.0752 (0.46)	0.1740 (0.27)
Speed variability, %	13.46 (4.20)	16.99 (5.63)	19.08 (8.69)	0.0152 (0.73)	0.2024 (0.28)	**0.0022 (0.83)**
ROM variability, %	10.56 (6.01)	13.12 (7.20)	20.42 (13.14)	0.1999 (0.39)	**0.0028 (0.69)**	**0.0004 (0.97)**
Flexion variability, %	24.57 (13.41)	42.78 (23.39)	59.46 (33.79)	**0.0014 (0.97)**	**0.0099 (0.58)**	**<0.0001 (1.39)**
Counted numbers, n	20.91 (8.25)	16.15 (7.86)	11.14 (7.07)	**0.0032 (0.59)**	**0.0275 (0.67)**	**<0.0001 (1.28)**
Counting mistakes, n	1.80 (2.00)	2.59 (1.99)	2.05 (1.76)	0.0879 (0.40)	0.2835 (0.29)	0.5767 (0.13)

UEF: upper-extremity function; ANOVA: analysis of variance; ROM: range of motion.

CN: cognitive normal; MCI: mild cognitive impairment; AD: Alzheimer’s disease.

Comparing dual-task trials between normal and rapid flexion condition, between groups differences in motor function agility were more pronounced within rapid elbow flexion (average effect size = 0.68 ± 0.33) compared to normal speed flexion (average effect size = 0.41 ± 0.34). On the other hand, flexibility of flexion and motor function variability were more noticeably different between cognitive groups when participants were asked to do the UEF test as consistently as possible (average effect size = 0.65 ± 0.29 for flexibility and average effect size = 0.77 ± 0.33 for variability) rather than rapidly (average effect size = 0.37 ± 0.16 for flexibility and average effect size = 0.41 ± 0.30 for variability).

Among dual-task cost parameters, rise time, flexion number, and flexion variability showed significant between group differences within the cognitive task of counting backward by threes (average effect size = 0.49 ± 0.37 and 0.44 ± 0.37 for normal and rapid elbow flexion, respectively). Overall, similar to our previous findings^17^, we observed higher effect sizes and less between-subject variability within dual-task trials compared to dual-task costs, and therefore, we used dual-task variables for further UEF cognitive index development.

### UEF cognitive index

Based on graph inspections and Shapiro-Wilk tests, UEF parameter distributions appeared normal, with the exception of rise time, for which logarithmic transformation was used to provide normal distribution. Within rapid flexion and counting backward by threes, univariate logistic models revealed that all UEF parameters were significantly associated with the cognitive status (*p* < 0.03), except for ROM (*p* = 0.46). For rapid flexion with the easier cognitive task of counting backward by ones, in addition to ROM, speed and flexion variability were also not significantly associated with the cognitive status (*p* > 0.15). Within the normal flexion condition, for both cognitive task difficulty, all UEF variables were significantly associated with cognition status (*p* < 0.001), except for ROM and speed parameters (*p* > 0.06).

A forward stepwise approach for selection of UEF parameters as independent variables resulted in flexion number and ROM variability as included parameters under rapid flexion condition, and additionally flexion variability under normal self-selected pace flexion condition (Table 5). Age, gender, and BMI, as well as parameters related to cognitive task performance were not included in the models, since, in addition to UEF parameters, they were not significantly associated with the cognitive status. Using the model with the whole sample, highest values for receiver operating characteristic (ROC) area under curves were achieved within normal self-selected pace UEF (ROC area under curve of 0.83 in predicting MCI and AD for both cognitive conditions, Table 5). Accordingly, we performed the 10-fold cross validation only for UEF normal speed tests. Results from 10-fold cross-validation for normal pace counting backward by ones showed mean values of 0.83 ± 0.01 and 0.83 ± 0.02 for ROC area under curve for MCI and AD prediction within the training sets, and sensitivity and specificity of 0.77 ± 0.17 and 0.71 ± 0.23 for predicting cognitive impairment (MCI and early AD) within the testing sets, respectively. Corresponding values were 0.83 ± 0.03 and 0.82 ± 0.03, 0.80 ± 0.21 and 0.75 ± 0.21 for normal pace elbow flexion while counting backward by threes. Using the ordinal logistic regression models, two probability equations (categorical cognitive indexes) were derived for each of normal pace UEF tests, from parameter estimates (Supplementary Information 1). Similarly, two UEF cognitive scores were established for each normal self-selected pace UEF tests (Supplementary Information 2). Results showed significant correlation between the UEF cognitive score with both MoCA and MMSE (*p* < 0.0001, *r* = 0.61 for MoCA and *r* = 0.42 for MMSE on average).

**Table 5:** Multivariable ordinal logistic UEF models for two flexion speeds and two cognitive task difficulties. A significant independent association between UEF parameters and cognitive status is highlighted in bold. Sensitivity and specificity values are indicated for cognitive impairment predictions (MCI and AD groups combined).

Independent variables	Parameter estimates	Standard errors	Chi-square (χ^2^)	*p*-value (95% CI)
**Rapid flexion counting backward by ones** (AIC = 180.29; Sensitivity = 0.77; Specificity = 0.54)				
MCI prediction: ROC area under curve = 0.72				
AD prediction: ROC area under curve = 0.80				
Intercept, [CN]	−3.6674	0.9786	14.04	**0.0002**
Intercept, [MCI]	−1.6965	0.9143	3.44	0.0635
Flexion number, n	0.2004	0.0484	17.13	**<0.0001 (0.1112,0.3016)**
ROM variability, %	−0.0650	0.0317	4.21	**0.0402 (−0.1305,−0.0020)**
**Rapid flexion counting backward by threes** (AIC = 167.06; Sensitivity = 0.70; Specificity = 0.57)				
MCI prediction: ROC area under curve = 0.79				
AD prediction: ROC area under curve = 0.78				
Intercept, [CN]	−2.1434	0.6835	9.83	**0.0017**
Intercept, [MCI]	0.0381	0.6406	0.00	0.9526
Flexion number, n	0.1669	0.0383	19.01	**<0.0001 (0.0966,0.2479)**
ROM variability, %	−0.0553	0.0241	5.27	**0.0217 (−0.1050,−0.0141)**
**Normal self-selected pace flexion counting backward by ones** (AIC = 172.37; Sensitivity = 0.72; Specificity = 0.72)				
MCI prediction: ROC area under curve = 0.83				
AD prediction: ROC area under curve = 0.83				
Intercept, [CN]	−1.2051	0.8250	2.13	0.1441
Intercept, [MCI]	0.9933	0.8334	1.42	0.2333
Flexion number, n	0.0530	0.0177	8.98	**0.0027 (0.0202,0.0894)**
ROM variability, %	−0.0911	0.0388	5.50	**0.0190 (−0.1720,−0.0159)**
Flexion variability, %	−0.0277	0.0109	6.49	**0.0109 (−0.0497,−0.0077)**
**Normal self-selected pace flexion counting backward by threes** (AIC = 164.87; Sensitivity = 0.82; Specificity = 0.70)				
MCI prediction: ROC area under curve = 0.83				
AD prediction: ROC area under curve = 0.83				
Intercept, [CN]	−1.5121	1.0668	2.01	0.1564
Intercept, [MCI]	0.7976	1.0665	0.56	0.4546
Flexion number, n	0.0838	0.0280	8.93	**0.0028 (0.0321,0.1431)**
ROM variability, %	−0.0446	0.0294	2.31	0.1288 (−0.1069,0.0128)
Flexion variability, %	−0.0202	0.0118	2.93	0.0870 (−0.0445,0.0025)

UEF: upper-extremity function; AIC: Akaike information criterion; MCI: mild cognitive impairment.

AD: Alzheimer’s disease; ROC: receiver operating characteristic; ROM: range of motion.

## Discussion

### UEF cognitive index for screening alzheimer’s disease

As hypothesized, we were able to discriminate MCI and AD with maximum sensitivity and specificity of 0.82 and 0.72 (ROC area under curve = 0.83). Within our sample, MMSE provided a sensitivity and specificity of 33% and 94% for identifying the combined group of MCI and AD, using a previously established cutoff of below 24 for cognitive impairment. Interestingly, when only AD participants were considered as the cognitively impaired group, sensitivity and specificity of 100% and 38% were achieved. These findings were in agreement with previous work suggesting MMSE provide better sensitivity in identifying more progressed stages of cognitive impairment, rather than MCI^28^. Using MoCA with a cutoff of below 26 for cognitive impairment, within our sample, combined groups of MCI and AD were identified with a sensitivity and specificity of 96% and 67%. The sensitivity of MoCA increased to 100% when only AD participants were considered as the cognitively impaired group; while specificity dropped to 38%. These values of sensitivity and specificity were similar but lower than previously reported values for MoCA, which were 90% sensitivity and 87% specificity^27^. Overall, these findings suggest that MoCA provides a very sensitive and rather specific tool for screening MCI and early AD. Comparing UEF with MMSE, UEF may be advantageous in discriminating between MCI and CN. Further, UEF prediction accuracy was found to be comparable to MoCA for identifying MCI and AD.

Among several parameters related to motor performance the agility, measured by the flexion number, and the variability, measured by ROM and flexion variability were the only predicting parameters included in cognitive indexes. This is in agreement with our previous findings^17,18^, as well as previous research on gait dual-task; gait variability and stride velocity within the dual-task condition have been reported as the most sensitive parameters for assessing cognitive impairment, compared to other spatial-temporal gait parameters^12,38,40,47^.

Results from ANOVA and logistic models showed that dual-task performance provides a better distinction of cognitive status compared to dual-task cost, especially when performing normal self-selected pace testing. The calculation of dual-task cost was included here to account for musculoskeletal deficits, by normalizing the dual-task performance for each participant using the single-task baseline. However, dual-task cost parameters could not discriminate as well here, and therefore, only dual-task parameters were included in the UEF cognitive models. We believe performing normal speed dual-task instead of rapid elbow flexion could minimize the influence of musculoskeletal deficits on cognitive task performance. Further, the main goal of this study was to develop a clinically “quick” screening tool for cognitive assessment. Accordingly, employing an index that involves only one 60-second trial (instead of two 60-second trials) would be advantageous for busy clinical settings. Also, unlike walking that can be excessively influenced by muscle strength, reflexive performance, and dynamic balance deficits, elbow flexion is a less musculoskeletal demanding task, which may be more suitable for cognitive assessment.

Parameters related to the performance of the secondary task were not selected in the index model because they did not improve models of cognitive status with motor performance. Although results also showed significant differences in cognitive task performance among cognitive groups, for between-group comparisons, higher effect sizes were achieved for UEF motor task parameters (Tables 3 and 4).

### The effect of motor and cognitive task conditions

Interestingly, unlike what was hypothesized, increasing the cognitive task difficulty did not noticeably influence the cognitive status predictions, especially within normal self-selected pace of elbow flexion. Although, within rapid motor task function, increasing the cognitive task difficulty slightly enhanced the UEF index cognitive prediction (by 4%), this influence was not observed within normal speed elbow flexion. Previous studies suggested that in dual-task testing the cognitive task should be sufficiently challenging to bring individuals near the limit of their ability^48^. Within this study, for the first time, we investigated the execution of an uncommon motor function and asked participants to perform this task as consistently as possible. Results from the current study suggest performing an uncommon motor task (rather than a motor task that is performed on daily basis such as walking), may require some skill-learning factors and consequently provide higher degrees of brain cortex challenges^49^. Accordingly, working memory may primarily get involved in learning this new motor task as well as counting numbers^50,51^, and therefore, even a simpler task of counting backward by ones could push MCI and early AD participants to show motor function deficits within dual-tasking. Nonetheless, the hypothesis of skill-learning factors within UEF should be confirmed in future research using brain imaging evidence (e.g., fMRI).

Overall, asking participants to perform the motor task consistently, rather than rapidly, improved the MCI and AD predictions by roughly 9%. Due to the imposed risk of falling within the rapid gait, especially when it is combined with a cognitive task, limited research exists to investigate differences in dual-task performance with respect to motor task execution speed in gait studies^52^. More specifically, to the best of our knowledge, no study exists to compare the quality of cognitive impairment predictions in older adults using both normal and rapid speed walking^53,54^. Current findings, for the first time, provide evidence that performing dual-task consistently with a desired speed can be advantageous over rapid motor task execution for cognitive impairment assessment among elders.

### Limitations and future direction

Several confounding parameters, including age, depression, comorbidity, and physical frailty were considered for adjustment within the current study. However, other potential confounding variables inherently may exist for performing UEF as a cognitive screening tool, such as the level of education and severe elbow arthritis. Within the current approach, an index was developed that only relies on motor function performance, and therefore, minimizes the influence of education level in cognitive screening. Further, previous research and our previous UEF motor function assessment showed most elders with arthritis are still able to perform UEF as it involves elbow flexion (rather than other joints prone to injury such as shoulder)^15^.

Due to the selection criteria, the generalizability of the current findings is limited to MCI of the Alzheimer’s type and early AD (groups chosen to reduce between-subject variability within our small sample size). However, as AD is the most common type of dementia, we believe current findings could provide a promising screening tool for assessing cognitive impairment in most older adults, with potential future use for other dementias. In follow-up studies to the current study, we will address this limitation by recruiting participants with other definitively diagnosed types of dementia.

Also, the current study lacks intra- and inter-rater reliability assessments; however, UEF was tested four times within the current experimental setup, and consistent results were observed within ANOVA models and UEF index development. Further, within several studies, the UEF motor task has been validated for frailty assessment among larger samples of older adults within different experimental settings. Of note, in continuation of this research, we will incorporate the UEF cognitive index into the original UEF frailty score.

Within the current study the UEF is validated to provide a sensitivity and specificity of 0.82 and 0.72, respectively. Although the specificity of UEF for identifying AD is not high, we believe, for the purpose of screening AD, UEF would provide acceptable sensitivity. Although acceptable sensitivity was achieved using UEF parameters and findings support consistency in current findings, the accuracy of cognitive status prediction may further be improved using dynamic analysis of elbow flexion motion. Within gait trials, several approaches have been implemented previously for regularity (repeatability) and stability assessment of dynamic motion within gait trials. The same approaches can be implemented within the current setup, which will be addressed in our future studies.

Finally, the association between UEF cognitive score (and its components) with neuropsychological tests has not been investigated here. In future research, we will assess the association between the developed UEF cognitive index with different components of cognitive impairments within neuropsychological tests, including working memory, attention, and executive functioning deficits within our cohort.

## Conclusion

Within the current study, for the first time, we assess the association between UEF dual-task among elders with clinically diagnosed cognitive impairments. Findings suggest that both speed and accuracy of motor function performance provide physiologically meaningful outcomes for screening cognitive impairment among older adults, especially when individuals are asked to perform the motor function as consistently as possible. Further, within the current sample, no noticeable difference was observed in predicting the cognitive status by implementing more challenging cognitive task (counting backward by threes versus ones). Overall, the current study showed promise in implementing UEF cognitive index as a simple and quick tool for screening cognitive impairment, which can make the motor function task less demanding and more suitable for elders with mobility impairments.

## Supplementary information

UEF categorical cognitive index and UEF cognitive score
