# Supplementary material for: Screening older adults for amnestic mild cognitive impairment and early-stage Alzheimer’s disease using upper-extremity dual-tasking
Source: Sci Rep. 2019 Jul 29;9:10911. doi: 10.1038/s41598-019-46925-y (PMC6662814; doi:10.1038/s41598-019-46925-y)
Supplement: Supplementary file 1 — UEF categorical cognitive index and UEF cognitive score [file 41598_2019_46925_MOESM1_ESM.pdf]

**Title**: Screening older adults for amnestic mild cognitive impairment and early-stage Alzheimer's disease using upper-extremity dual-tasking

*Nima Toosizadeh*[* 1,2,3]*, Hossein Ehsani*[1]*, Christopher Wendel*[2,3]*, Edward Zamrini*[4,5,6]*, Kathy O'Connor*[4,5]*, Jane Mohler*[1,2,3]

1: Department of Biomedical Engineering, University of Arizona, Tucson, AZ, USA

2: Arizona Center on Aging (ACOA), Department of Medicine, University of Arizona, College of Medicine, Tucson, AZ, USA

3: Division of Geriatrics, General Internal Medicine and Palliative Medicine, Department of Medicine, University of Arizona, Tucson, AZ, USA

4: Banner Sun Health Research Institute, Sun City, AZ, USA

5: Banner Alzheimer's Institute, University of Arizona, Tucson, AZ, USA

6: Department of Neurology, University of Utah, Salt Lake City, UT, USA

Corresponding Author:
Nima Toosizadeh
Arizona Center on Aging (ACOA)
Department of Biomedical Engineering
University of Arizona, College of Engineering
Bioscience Research Lab (BSRL)
1601 E Helen St., Tucson AZ, 85719
Telephone: (+1) 520-444-5265
Fax: (+1) 520-626-5811
Email: ntoosizadeh@aging.arizona.edu

## Supplementary Information 1 – UEF categorical index

To construct models, the following steps were followed: 1) descriptive analysis of UEF parameters: outlier detection using box plots and histograms and testing of distribution normality using Shapiro-Wilk *W* test; 2) univariate analysis: UEF parameters with significant association with cognitive status were selected for subsequent steps; 3) testing of collinearity between UEF parameters: using variance inflation factor (VIF) values. A VIF cutoff value larger than 10 was considered an indication of collinearity presence [50]; 4) stepwise parameter selection: UEF and demographic parameters were selected based on Akaike information criterion (AIC) values; and 5) model evaluation: using 10-fold cross-validation, in which the sample was randomly divided into k = 10 equal parts, with the same number of participant assignment in each cognitive group. At each $k^{th}$ iteration, k − 1 partitions were used as the training dataset and the left out partition was used as the validation dataset. The mean and standard deviation across training sets were calculated for area under receiver operating characteristic (ROC) curves, AIC within training sets, and sensitivity and specificity within testing sets were calculated. For UEF categorical cognitive index, the probability equations were derived using parameter estimates ($\beta_i$) and magnitude of independent variables ($a_i$) as follows. Two UEF cognitive categorical indexes were derived for normal self-selected pace elbow flexion within two cognitive conditions of counting backward by ones and threes (Table S1).

$$P[CN] = \frac{1}{1+\exp -(\beta_1+\beta_3 a_3+\beta_4 a_4+\beta_5 a_5)} \quad (A1)$$

$$P[MCI] = \frac{1}{1+\exp -(\beta_2+\beta_3 a_3+\beta_4 a_4+\beta_5 a_5)} - \frac{1}{1+\exp -(\beta_1+\beta_3 a_3+\beta_4 a_4+\beta_5 a_5)} \quad (A2)$$

$$P[AD] = 1 - \frac{1}{1+\exp -(\beta_2+\beta_3 a_3+\beta_4 a_4+\beta_5 a_5)} \quad (A3)$$

Table S1: UEF categorical cognitive index, Independent variables ($a_i$) and parameter estimates ($\beta_i$) are presented

| **Independent Variables** | **Parameter Estimates** | |
|---|---|---|
| | **Normal self-selected pace counting backward by ones** | **Normal self-selected pace counting backward by threes** |
| Intercept, [CN] | $\beta_1$ = -1.2051 | $\beta_1$ = -1.5121 |
| Intercept, [MCI] | $\beta_2$ = 0.9933 | $\beta_2$ = 0.7976 |
| $a_3$: Flexion number, n | $\beta_3$ = 0.0530 | $\beta_3$ = 0.0838 |
| $a_4$: ROM variability, % | $\beta_4$ = -0.0911 | $\beta_4$ = -0.0446 |
| $a_5$: Flexion variability, % | $\beta_5$ = -0.0277 | $\beta_5$ = -0.0202 |

CN: Cognitive normal
MCI: mild cognitive impairment
AD: Alzheimer's disease
ROM: range of motion

## Supplementary Information 2 – UEF score

To develop the UEF cognitive score, parameters selected from the categorical index were used. First, each continuous UEF parameter were split into three categories based on mean values for each cognitive group. That is parameter mean values were considered as reference values (W) and the mid-points between reference values were considered as cut-offs. Next, the distance between each category and the lowest (reference) category in regression units was determined, by multiplying the β (parameter estimates from the categorical index model) by the difference between the category W and the reference category $W_{REF}$, or β ($W-W_{ref}$). Then, each categorical independent variable was assigned a point value by dividing the distance β ($W-W_{ref}$) by a base constant that represents a coefficient value corresponding to one point (flexion number was selected here since it provided the smallest distance β($W-W_{ref}$)). Point values were rounded to the nearest integer. The UEF cognitive score (from 0: CN to 1: AD) for a given participant was determined by the sum of points corresponding to performance results from UEF dual-task test and the maximum possible points ([maximum – total]/total) (see Supplementary Information 1 for details regarding UEF categorical cognitive index). Two UEF cognitive scores were derived for normal self-selected pace elbow flexion within two cognitive conditions of counting backward by ones and threes.

Counting backward by ones (0: CN - 1: AD):

UEF cognitive score: The total score = (16 - total point) / 16

| Variable | Variable Ranges | Points (0-16) | Parameter Estimate (β) | Reference Value (W) | $\beta (W-W_{ref})$ | Points $\beta (W-W_{ref}) / B_{ref}$ † |
|---|---|---|---|---|---|---|
| Flexion number, n | < 35 | 0 | 0.053 | 34.23 | 0 | 0 |
| | 35 - 42 | 1 | | 37.09 | 0.15158 | 1 |
| | >42 | 5 | | 47.31 | 0.69324 | 4.573426573 |
| ROM variability, % | < 10 | 4 | -0.0911 | 8.40 | 0 | 0 |
| | 10 - 13 | 2 | | 10.64 | -0.204064 | -1.346246207 |
| | > 13 | 0 | | 14.6 | -0.56482 | -3.726217179 |
| Flexion variability, % | < 19 | 7 | -0.0277 | 11.50 | 0 | 0 |
| | 19 - 37 | 4 | | 25.58 | -0.390016 | -2.573004354 |
| | > 37 | 0 | | 47.57 | -0.999139 | -6.59149624 |

CN: Cognitive normal
MCI: mild cognitive impairment
AD: Alzheimer's disease
ROM: range of motion

Counting backward by threes (0: CN - 1: AD):

UEF cognitive score: The total score = (22 - total point) / 22

| Variable | Variable Ranges | Points (0-22) | Parameter Estimate (β) | Reference Value (W) | $\beta (W-W_{ref})$ | Points $\beta (W-W_{ref}) / B_{ref}$ † |
|---|---|---|---|---|---|---|
| Flexion number, n | < 22 | 0 | 0.0838 | 19.73 | 0 | 0 |
| | 22 - 30 | 3 | | 23.88 | 0.34777 | 3.045911575 |
| | > 30 | 12 | | 35.97 | 1.360912 | 11.91942265 |
| ROM variability, % | < 12 | 4 | -0.0446 | 10.56 | 0 | 0 |
| | 12 and 17 | 3 | | 13.12 | -0.114176 | -1 |
| | > 17 | 0 | | 20.42 | -0.439756 | -3.8515625 |
| Flexion variability, % | < 34 | 6 | -0.0202 | 25.57 | 0 | 0 |
| | 34 - 51 | 3 | | 42.78 | -0.347642 | -3.044790499 |
| | > 51 | 0 | | 59.46 | -0.684578 | -5.995813481 |

CN: Cognitive normal
MCI: mild cognitive impairment
AD: Alzheimer's disease
ROM: range of motion
